# Urinary Biomarkers of Oxidative Stress in Aging: Implications for Prediction of Accelerated Biological Age in Prospective Cohort Studies

**DOI:** 10.1155/2022/6110226

**Published:** 2022-05-06

**Authors:** Peter Mukli, Dee H. Wu, Tamas Csipo, Cameron D. Owens, Agnes Lipecz, Frigyes Samuel Racz, Fouad A. Zouein, Adam Tabak, Anna Csiszar, Zoltan Ungvari, Panayiotis D. Tsitouras, Andriy Yabluchanskiy

**Affiliations:** ^1^Vascular Cognitive Impairment and Neurodegeneration Program, Oklahoma Center for Geroscience and Healthy Brain Aging, Department of Biochemistry and Molecular Biology, University of Oklahoma Health Sciences Center, Oklahoma City, OK, USA; ^2^Department of Physiology, Semmelweis University, Budapest, Hungary; ^3^Department of Radiological Sciences, University of Oklahoma Health Sciences Center, Oklahoma City, OK, USA; ^4^The Peggy and Charles Stephenson Cancer Center, University of Oklahoma Health Sciences Center, Oklahoma City, OK, USA; ^5^International Training Program in Geroscience, Doctoral School of Basic and Translational Medicine/Department of Public Health, Semmelweis University, Budapest, Hungary; ^6^Department of Neurology, Dell Medical School, University of Texas at Austin, Austin, TX, USA; ^7^The Cardiovascular, Renal, and Metabolic Diseases Research Center of Excellence, American University of Beirut Medical Center, Riad El-Solh, Beirut, Lebanon; ^8^Department of Signaling and Cardiovascular Pathophysiology, UMR-S 1180, Inserm, Université Paris-Saclay, France; ^9^Department of Pharmacology and Toxicology, School of Medicine, University of Mississippi Medical Center, Jackson, MS, USA; ^10^1st Department of Medicine, Faculty of Medicine, Semmelweis University, Budapest, Hungary; ^11^International Training Program in Geroscience, Doctoral School of Basic and Translational Medicine/Department of Translational Medicine, Semmelweis University, Budapest, Hungary; ^12^Department of Health Promotion Sciences, College of Public Health, University of Oklahoma Health Sciences Center, Oklahoma City, OK, USA

## Abstract

**Background:**

Aging is a major risk factor for a range of chronic diseases. Oxidative stress theory of aging has been previously proposed as one of the mechanisms responsible for the age-related decline in organ/tissue function and the development of age-related diseases. Urine contains rich biological information on the health status of every major organ system and can be an important noninvasive source for biomarkers of systemic oxidative stress in aging.

**Aims:**

The objective of this cross-sectional study was to validate a novel panel of urinary oxidative stress biomarkers.

**Methods:**

Nucleic acid oxidation adducts and oxidative damage markers of lipids and proteins were assessed in urine samples from nondiabetic and currently nonsmoking subjects (*n* = 198) across different ages (20 to 89 years old). Urinary parameters and chronological age were correlated then the biological age of enrolled individuals was determined from the urinary oxidative stress markers using the algorithm of Klemera and Doubal.

**Results:**

Our findings showed that 8-oxo-7,8-deoxyguanosine (8-oxoG), 8-oxo-7,8-dihydroguanosine (8-OHdG), and dityrosine (DTyr) positively correlated with chronological age, while the level of an F_2_-isoprostane (iPF_2_*α*-VI) correlated negatively with age. We found that 8-oxoG, DTyr, and iPF_2_*α*-VI were significantly higher among accelerated agers compared to nonaccelerated agers and that a decision tree model could successfully identify accelerated agers with an accuracy of >92%. *Discussion*. Our results indicate that 8-oxoG and iPF_2_*α*-VI levels in the urine reveal biological aging.

**Conclusion:**

Assessing urinary biomarkers of oxidative stress may be an important approach for the evaluation of biological age by identifying individuals at accelerated risk for the development of age-related diseases.

## 1. Introduction

Advancing age is a major risk factor for a range of chronic diseases, including cardiovascular and cerebrovascular diseases, cognitive impairment and dementia, and cancer [1–4]. The oxidative stress theory of aging, originally proposed by Harman in 1956 [5], postulates that age-associated decline in cellular functions and, by extension, the pathogenesis of age-related diseases are caused by increased production of reactive oxygen species (ROS) and a consequential accumulation of oxidative damage to macromolecules (proteins, nucleic acids, and lipids) [6, 7]. Although this theory has been repeatedly challenged over the decades as the aging phenomena cannot be simplified to a manifestation of accumulating oxidative damage, ROS are shown to play a critical role in diverse cellular processes of aging and contributes to physiological deterioration in various organ systems, including the cardiovascular system [1, 8–14].

Modern geroscience research has established that shared, evolutionarily conserved cellular and molecular mechanisms of aging do exist and contribute to the genesis of age-related diseases. These synergistic processes of aging (referred to as the “pillars of aging”) have been mechanistically linked, either directly or indirectly, to increased oxidative stress. Pillars of aging include increased inflammation, epigenetic changes, loss of proteostasis, altered metabolism, impaired stem cell regeneration, decreased adaptation to stress, and macromolecular damage [15]. Prospective human studies of aging and age-related diseases [16, 17] have suggested that individuals age at different rates. The picture has emerged that in every population, there are individuals with advanced biological age (accelerated agers) having poorer physical function and cognitive performance compared to a representative age-matched reference sample. Accelerated agers, whose biological age exceeds their chronological age (CA), present with age-related diseases earlier in life than those individuals with the same CA. Understanding the underlying causes for heterogeneity in health and morbidity of older adults is a fundamental question in geroscience research. Given the vital role of ROS in various biological processes of aging and the pathogenesis of age-related diseases, it is essential to assess biomarkers of oxidative stress in prospective human studies of aging.

Measurement of net antioxidant capacity of serum and circulating biomarkers of oxidative stress are available for use in human studies, including assays of circulating isoprostanes, oxidative protein modifications, oxidized low-density lipoprotein, and oxidized phospholipids [18–20]. Searching for sensitive and noninvasive biomarkers, however, has been a major challenge for geroscience research. Urine provides a convenient biospecimen for analyzing age-related biomarkers and can also be collected recurrently from older adults (in whom compliance with invasive procedures is a major issue) noninvasively and in large volumes. Urine contains rich and multifaceted biological information on the health status of every major organ system and may potentially be an important and noninvasive source for biomarkers of systemic oxidative stress in aging [21–26]. Yet, despite their importance, urinary biomarkers have been underutilized in geroscience research when investigating the mechanisms responsible for the heterogeneity of aging and the pathogenesis of age-related diseases.

The present study was designed to provide initial validation for a novel panel of urinary biomarkers of oxidative stress with aging. In a cross-sectional study of 198 individuals, urinary markers of macromolecular oxidative damage were assessed in urine samples—including 8-oxo-7,8-deoxyguanosine (8-oxoG), 8-oxo-7,8-dihydroguanosine (8-OHdG), dityrosine (DTyr), F_2_-isoprostanes (iPF_2_*α*-III, iPF_2_*α*-VI), and 2,3-dinor-8-iso-prostaglandin F_2_*α* (Dinor)—from nondiabetic and currently nonsmoking subjects across different ages (20 to 89). To describe heterogeneity in aging, the BA of these individuals was determined using the urinary oxidative stress markers based on the algorithm proposed by Klemera and Doubal [27]. The ability of BA calculation for the identification of accelerated aging was assessed by classifying each subject with the aid of a decision tree that uses a constellation of urinary biomarkers of oxidative stress.

## 2. Materials and Methods

### 2.1. Study Participants' Characteristics

This study was performed on urine and blood samples collected from 198 adults (103 females and 95 males) of 20–89 years of chronological age, never smokers or ex-smokers (denied use of tobacco in the previous 3 years) as described in Ref [28]. The research protocol was approved by the Arizona State University Institutional Review Board, and subjects gave written informed consent to participate. All subjects provided informed consent prior to participation in the study [28]. To minimize confounding bias affecting apparent biological aging, subjects with active cancer, chronic cardiovascular, renal or neurodegenerative diseases, prediabetic and diabetic states, and with BMI < 20 or BMI > 30 were excluded from the analysis. Individuals with a high fasting blood glucose level (>6.0 mmol/l) or with elevated hemoglobin A1c (≥6%), indicating the average level of hemoglobin glycosylation in the preceding 8-12 weeks, were also excluded. A total of 131 subjects were selected for further analyses. Characteristics of included participants are shown in Table 1.

### 2.2. Laboratory Evaluation

For urinalysis of oxidative stress markers, the primary outcome parameters, a first-morning specimen was obtained from each participant. Aliquots of urine were frozen to a temperature of −80°C immediately after their collection, and measurements were performed on defrosted and centrifuged urine samples as described previously [28, 29]. The concentration of all compounds was determined by high-pressure liquid chromatography (HPLC)/tandem mass spectrometry (MS). The HPLC system consisted of three Shimadzu LC-10 AD pumps, a Shimadzu degasser (Shimadzu Scientific Instruments, Columbia, MD, USA), and a Perkin Elmer autosampler (Perkin Elmer LLC, Norwalk, CT, USA). Briefly, for all oxidative damage adducts, 10 *μ*l of standard or urine samples was spiked and then injected onto a YMC ODS-AQ column (2.0 × 50 mm, 3 *μ*m particle size; Waters, Milford, MA, USA) with an identical guard column (2.0 × 10 mm, 3 *μ*m). The sample was delivered at a flow rate of 200 *μ*l/min. In the case of oxidized nucleosides and dityrosine assessment, the mobile phase consisted of 10 mM ammonium acetate, formic acid (A_1_), and methanol (B_1_). Subsequently, these components were separated between 2 and 7.5 min of HPLC running time by using a solvent gradient program (95% A_1_ at time 0, a linear decrease to 50% A_1_ at 6.0 min, hold for 30 s, drop to 0% A_1_ within 30 s, then increase from 0 to 95% A_1_ within 1 min) and then injected into the MS. To detect 8-oxoG and 8-OHdG, multiple reaction monitoring (MRM) mode was used with ion pairs (*m*/*z*) of 300/168 284/168, respectively; while DTyr was detected in MRM mode with positive ionization. For more details of the procedure, see Ref. [28]. The samples used for assessment of iPF_2_*α*-III, iPF_2_*α*-VI, and Dinor were dissolved in a mobile phase consisting of methanol : acetonitrile (5 : 95 v/v) (A_2_) and 2 mM ammonium acetate (B_2_). These components were separated between 3 and 8 minutes of HPLC running time by using a solvent gradient program (15% A_2_ at time 0, a linear increase to 70% A_2_ at 6 min, a linear increase to 100% A_2_ at 8 min, then a linear decrease from 100 to 15% A_2_ within 1 min) and then injected into the MS. The MRM pairs for detecting iPF2*α*-III, iPF2*α*-VI, and Dinor were 353/193, 353/115, and 325/237, respectively; for further details, see Ref. [29].

Creatinine content of urine was determined using a commercially available clinical test kit with a chemistry analyzer system (Synchron Clinical System LX20; Beckman Coulter, Fullerton, CA, USA) [28] and was regarded as a confounder of the outcome measures. Hence, to normalize urinary oxidative stress markers, their measured concentrations (measured in ng/ml) were divided by urinary creatinine content (measured in *μ*g/g).

### 2.3. Calculation of Biological Age

Klemera and Doubal developed a mathematical model (KDM [27]) that estimates biological age based on selected variables that correlate with the chronological age for any sex. It is assumed that fluctuation of BA around CA is represented in the variation of any parameter that systematically changes with age (CA predictors). Hence, their difference is defined as a random variable (*R*_*BA*_) with mean zero and variance *s*_*AB*_^2^:

(1) $$\begin{array}{c} BA-CA=R_{AB}\left(0;s_{BA}^{2}\right). \end{array}$$

As KDM requires independent variables, principal component analysis was performed to obtain *m* = 6 predictors explaining >95% variability of CA, which also reduced the dimensionality of the data. The actual value of aging markers (*x*_*j*_) can be affected by transient random effects (with mean 0 and variance *S*_*j*_^2^) independent from BA that is described as

(2) $$\begin{array}{c} x_{j}=F_{j}\left(BA\right)+R_{j}\left(0;s_{j}^{2}\right). \end{array}$$

In the simplest case, a linear relationship is assumed with *F*_*j*_ between *x*_*j*_ and BA with a slope *k*_*j*_ and intercept (bias) *q*_*j*_: *x*_*j*_ = *k*_*j*_*BA* + *q*_*j*_. The most accurate estimate of BA uses CA as an aging biomarker and is given by the following equation:

(3) $$\begin{array}{c} \hat{BA}=\frac{\sum_{j=1}^{m}\left(x_{j}-q_{j}\right)\left(k_{j}/s_{j}^{2}\right)+\left(CA/s_{BA}^{2}\right)}{\sum_{j=1}^{m}{\left(k_{j}/s_{j}\right)}^{2}+\left(1/s_{BA}^{2}\right)}. \end{array}$$

The BA was calculated as the value corresponding to the minimal distance between regression lines in an *m-*dimensional predictor space, which is achieved by estimating slope, intercept, and variance parameters of the fitted regression model. In this study, we used all parameters of urine samples as initial independent parameters that were correlated with CA (inclusion criterium: *r* > 0.1 [30]). Biological age was then calculated using the TrueTrait function of the WGCNA R package, separately for men and women [31] (see the R script in the Appendix).

Finally, participants were assigned into groups of accelerated (*A*) or nonaccelerated (*N*) aging based on the difference between their BA and CA. The threshold value was set to 2.679 years, which is just sufficient to achieve a significant difference (*p* = 0.0500) in BA between such identified *A* and *N* groups.

### 2.4. Statistical Analysis

Statistical analyses were carried out using the Statistica 13.5 (TIBCO, Palo Alto, CA, USA) software. The normal distribution of data was evaluated using Shapiro-Wilk's test. The relationship between measures of urinary oxidative stress parameters (dependent variable) and CA was assessed by Pearson correlation. Linear regression analyses have been performed with CA, smoking, sex, and BMI, as predictors along with their first-order interactions. The displayed results are grouped by sex or past smoking history, and figures were created with the Gramm toolbox [32] implemented in MATLAB 2017 (MathWorks, MA, Natick, USA). For hypothesis testing, the threshold level of significance was set to 0.05. Following standard conventions, *p* and *β* denote the probability of type I and type II error, respectively, thus, the power of the statistical test is 1 − *β*.

To predict accelerated aging at an individual level, a decision tree analysis was performed using the “Data Mining” module of Statistica 13.5. *A* and *N* labels were the dependent variables in the decision tree model. Urinary oxidative stress markers and BMI were used as continuous predictors together with sex and smoking history (categorical predictors). The test error of the algorithm was evaluated using a 10-fold cross-validation scheme. In that, the dataset was split randomly into ten nonoverlapping subgroups, and in each iteration, nine of them were used for training and with the tenth as a test set for the decision tree (hyperparameters: minimum *n* of cases: 13, maximum *n* of nodes: 1000). Proportions of *A* and *N* cases were similar in all subgroups. Classifier performance was characterized by the average number of correctly identified and misclassified cases summarized in an average confusion matrix.

## 3. Results

### 3.1. Correlation of Urinary Oxidative Stress Markers with Chronological Age

**Figure 1:**
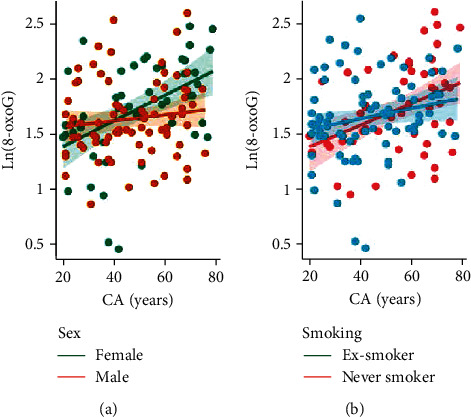
Relationship between chronological age (CA) and 8-oxo-7,8-deoxyguanosine (8-oxoG). 8-oxoG concentrations are normalized to urinary creatinine content, and their natural logarithmic values are shown (measurement unit: *μ*g/g). Ln(8-oxoG) significantly correlated with CA in females (*r* = 0.33, *p* = 0.016) but neither in males (*r* = 0.116, *p* = 0.306) (a); nor ex-smokers (*r* = 0.264, *p* = 0.056), nor never-smokers (*r* = 0.053, *p* = 0.645) (b).

**Figure 2:**
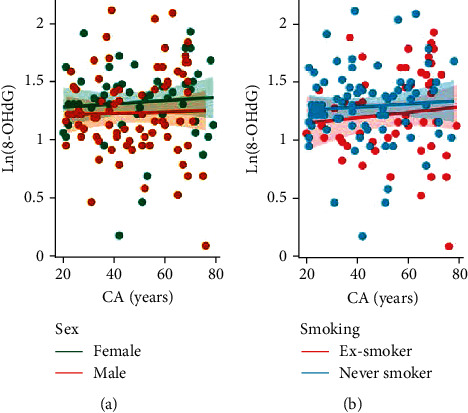
Relationship between chronological age (CA) and 8-oxo-7,8-dihydroguanosine (8-OHdG). 8-OHdG concentrations are normalized to urinary creatinine content, and their natural logarithmic values are shown (measurement unit: *μ*g/g). Ln(8-OHdG) significantly correlated with CA in females (*r* = 0.289, *p* = 0.037) but neither in males (*r* = 0.043, *p* = 0.700) (a); nor ex-smokers (*r* = 0.164, *p* = 0.239), nor never-smokers (*r* = 0.057, *p* = 0.619) (b).

We determined whether urinary markers of oxidative stress were useful predictors of CA by estimating the correlation between these variables. Urinary oxidative stress markers followed a log-normal distribution, which was considered in the linear regression analysis. The normalized urinary concentration of 8-oxoG, the main product of oxidative DNA damage, showed a moderate positive correlation with CA (*r* = 0.500 for females and *r* = 0.101 for males). This correlation depended on sex, indicated by a significantly steeper slope (*p* = 0.020) for female subjects (Figure 1(a)). The relationship for never and ex-smokers was not statistically different (Figure 1(b)). The normalized levels of 8-OHdG, an oxidant derived from RNA and excreted in the urine, showed a weak correlation with CA (*r* = 0.108 for females and *r* = 0.101 for males), which was not influenced by smoking history or sex (Figure 2).

**Figure 3:**
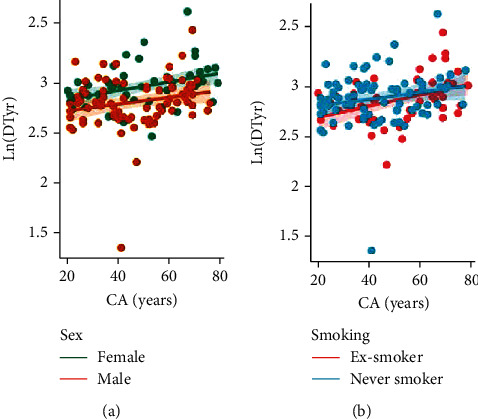
Relationship between chronological age (CA) and dityrosine (DTyr). DTyr concentrations are normalized to urinary creatinine content and their natural logarithmic values are shown (measurement unit: *μ*g/g). (a) Ln(DTyr) significantly correlated with CA both in females (*r* = 0.433, *p* = 0.001) and in males (*r* = 0.340, *p* = 0.002). (b) Ln(DTyr) significantly correlated with CA both in nonsmokers (*r* = 0.274, *p* = 0.015) and in ex-smokers (*r* = 0.441, *p* < 0.001).

**Figure 4:**
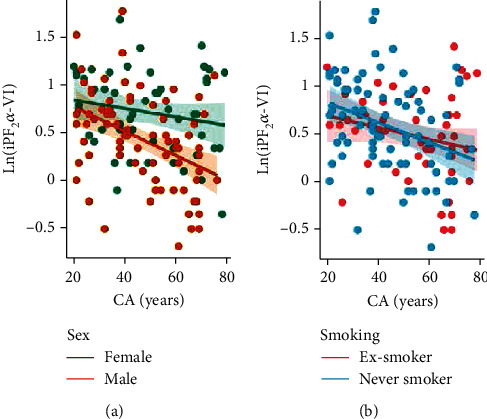
Relationship between chronological age and isoprostane-F_2_*α*-VI (iPF_2_*α*-VI). iPF_2_*α*-VI concentrations are normalized to urinary creatinine content, and their natural logarithmic values are shown (measurement unit: *μ*g/g). Ln(iPF_2_*α*-VI) did not correlate with CA regardless of sex (females: *r* = 0.006, *p* = 0.962; males: *r* = 0.122, *p* = 0.518) (a) or smoking history (never-smokers: *r* = 0.065, *p* = 0.567; ex-smokers: *r* = 0.098, *p* = 0.4838) (b).

Normalized urinary DTyr, a marker of oxidative protein damage, increased with chronological age but the positive correlation was not significant (*r* = 0.365, *p* = 0.231).  Figure 3 also shows that sex and smoking status did not alter this relationship. The only lipid peroxidation marker that showed correlation with CA was iPF_2_*α*-VI (Figure 4), indicated by *r* = −0.145 for females and *r* = −0.374 for males. In contrast, the linear relationship was much weaker for iPF_2_*α*-III and Dinor (data not shown), thus, these variables were excluded from BA calculation. The negative linear relationship is not explained by any of the examined predictors; however, BMI was significantly associated with the iPF_2_*α*-VI levels (*p* = 0.02, see Table 2).

It is of note that urinary creatinine content and measured concentrations of all urinary oxidative stress markers are inversely correlated with chronological age in both sexes (*p* < 0.05). Thus, it was necessary to obtain normalized values of oxidative damage adducts to take into account the bias due to age-related changes in kidney function.

### 3.2. Characteristics of the Accelerated Aging Group

**Figure 5:**
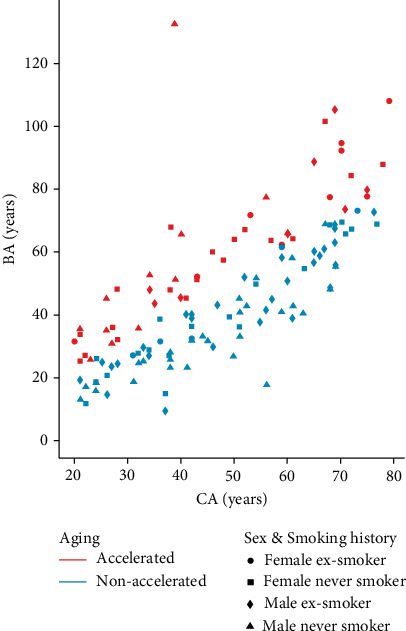
Relationship between biological age (BA) and chronological age (CA). Each dot represents BA and CA of one subject, color marks accelerated (*n* = 48) and nonaccelerated (*n* = 83) agers, symbol marks female never smokers (*n* = 38) and ex-smokers (*n* = 14), and male never smokers (*n* = 40) and ex-smokers (*n* = 39).

**Figure 6:**
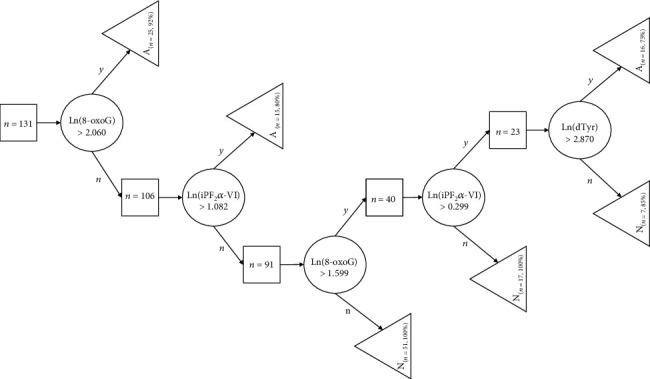
Decision tree for classifying accelerated aging. Squares represent nonterminal nodes with the number of corresponding cases (*n*). Each classified case appears in a terminal node denoted by a triangle (either *A*—accelerated agers or *N*—nonaccelerated ager cases). Decision rules based on thresholds for the given urinary oxidative stress marker levels and corresponding paths (yes/no) are illustrated by circles with branching arrows.

**Figure 7:**
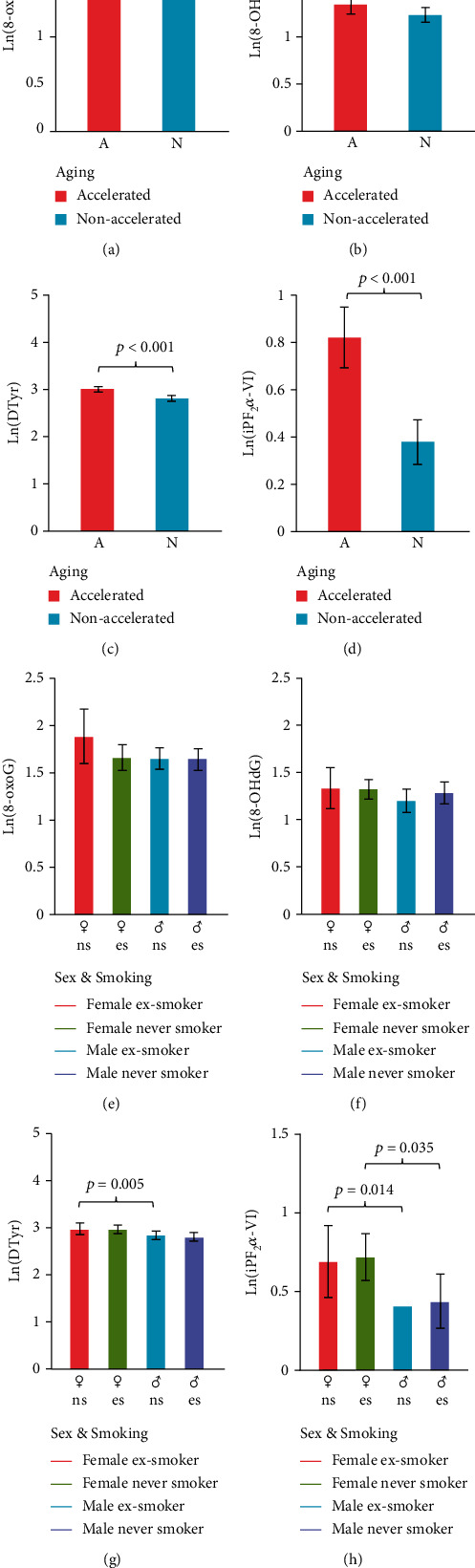
Group-level comparisons of urinary oxidative stress markers. Natural logarithm of concentrations was compared between accelerated (*n* = 48) and nonaccelerated (*n* = 83) agers (top panels, *t*-test) and across sexes and smoking history (bottom panels, see Table 1 for sample sizes; two-way ANOVA, Bonferroni post hoc test). (a, e) 8-oxo-7,8-deoxyguanosine (8-oxoG); (b, f) 8-oxo-7,8-deoxyguanosine (8-oxoG); (c, g) dityrosine (DTyr); (d, h) 8-oxo-7,8-deoxyguanosine (iPF_2_*α*-VI). Abbreviations: A: accelerated aging; N: nonaccelerated aging; ♀ns: female nonsmoker; ♀es: female ex-smoker; ♂ns: male nonsmoker; ♂es: male ex-smoker.

We calculated the BA using the KDM algorithm from important predictors of CA, including 8-OHdG, 8-oxoG, DTyr, and iPF_2_*α*-VI.  Figure 5 depicts the BA as a function of CA; cases of accelerated aging are marked with pink. A decision tree (Figure 6) was generated to classify between accelerated (*n* = 48) and nonaccelerated agers (*n* = 83) using urinary oxidate stress markers, smoking, sex, and BMI as features. The high classifier performance is justified by the number of misclassified and correctly identified cases (Table 3) which corresponded to an accuracy of 92.3%. Significantly higher levels were found in the accelerated ager group for 8-oxoG (7.1485 ± 1.3813 ng/ml vs. 4.5245 ± 1.3822, *t*-test for the log-transformed values: *df* = 129, *β* ≈ 0, Cohen's *d* = 1.90), DTyr (20.1459 ± 1.2761 vs. 16.6448 ± 1.2642, *t*-test, *df* = 129, *β* = 0, Cohen's *d*: 2.76), and iPF_2_*α*-VI (2.2733 ± 1.56261 vs. 1.4591 ± 1.5540, *t*-test, *df* = 129, *β* = 0.12, Cohen's *d*: 0.52) were significantly higher among accelerated agers compared to nonaccelerated agers, while these groups were not statistically different in terms of 8-OHdG concentration (Figure 7).

To evaluate the predictive capability of urinary oxidative stress markers, the analytical and statistical procedures were repeated on a comprehensive panel of serum parameters measured in venous blood samples (obtained after >10 hours of fasting). Specifically, BA was calculated from variables that correlate with CA with *r* > 0.1 from complete blood count and chemistry, including electrolytes, transport nutrients, lipids, proteins, hormones, antioxidants, vitamin-like compounds, metabolites, and waste products (see Supplementary Material (available here)). The deviation of the estimated BA from CA was much higher compared to BA estimation of urine samples, and there were no significant differences between the accelerated aging and nonaccelerated aging group. This indicates that urinary oxidative stress parameters are significantly better predictors of biological aging than blood chemistry parameters.

## 4. Discussion

In the present study, our aim was to evaluate whether urinary oxidative stress markers correlate with chronological age (CA) and investigate whether these markers can predict biological age (BA) and accelerated aging. Major findings of this study are threefold: (1) urinary biomarkers of oxidative stress 8-oxoG, 8-OHdG, and DTyr positively correlated with CA, while iPF_2_*α*-VI correlated negatively with CA; (2) 8-oxoG, DTyr, and iPF_2_*α*-VI were significantly higher among accelerated agers compared to non-accelerated agers; and (3) a decision tree model could successfully identify accelerated aging with an accuracy of >92%.

The oxidative stress theory of aging implies that increased macromolecular oxidative damage contributes to the pathogenesis of age-related diseases. Consistent with predictions based on the oxidative stress theory of aging, urinary biomarker levels of oxidative stress were elevated in subjects with accelerated aging when compared to nonaccelerated agers. The observed elevated urinary levels 8-oxoG and 8-OHdG, Dinor, and DTyr reflect a higher degree of oxidative damage of nucleic acids and proteins in older adults, respectively [33, 34]. As to iPF2, a lipid peroxidation product of arachidonic acid, it was higher in young patients and in accelerated agers compared to old (Figure 4) or nonaccelerated agers (Figure 6), respectively. Our findings on urinary oxidative stress biomarkers extend the results of previous studies demonstrating age-related increases in levels of similar markers but in the systemic circulation [35, 36]. Accordingly, increased circulating levels of biomarkers of oxidative stress were shown to associate with multiple age-related diseases, including cancer, diabetes mellitus [21], neurodegenerative diseases, and cardiovascular diseases, as well as pathological conditions characterized by ongoing inflammatory processes [37, 38]. On another note, nitrative stress—imposed by reactive derivatives of NO—contributes to a plethora of pathophysiological processes as well as aging [7, 39–41].

This study also found that concentrations of urinary biomarkers of oxidative stress varied considerably in older individuals. Thus, we attempted to incorporate this novel set of biomarkers in estimating the BA of healthy individuals in the present cohort. The concept of BA has been introduced to geroscience research and has been refined throughout the past decades [27]. A recent study comparing four common BA estimation methods concluded that the Klemera and Doubal mathematical model (KDM) provides the most accurate risk estimation for morbidity and mortality [42–44]. Thus, in the present study, BA was estimated by the KDM method using specific urinary biomarkers of oxidative stress that correlated with chronological age (CA).

These biomarkers have several advantages over circulating biomarkers in that they are noninvasive, can be used for cross-species comparison [45], quantitative, change at rate reflecting the rate of aging, are relevant for physiological dysfunction, reproducible, show significant difference between individuals, and monitor a basic mechanism of aging. Smoking was reported to be associated with higher levels of nucleic acid oxidation adducts, iPF_2_*α*, and DTyr in the urine [28]; thus, we excluded current smokers from the present analysis.

The data obtained in this study were used to build a decision tree to identify accelerated agers whose BA exceeded CA with a statistically defined threshold. Although 8-oxoG, 8-OHdG, DTyr, and iPF_2_*α*-VI levels were used for calculation of BA, urinary concentrations of 8-oxoG and iPF_2_*α*-VI were sufficient to classify each subject with >90% of accuracy. Ultimately, these were the most important predictors of accelerated aging according to feature importance analysis, while BMI, smoking, and gender were the least important.

The present study has several limitations, including the sample size and the number of predictor and confounder variables. A larger population would allow for statistical adjustment of more confounders and to implement a more accurate supervised machine learning model either for classifying accelerated aging, presence of disease, or other major clinical outcomes. To implement more effective interventions in healthy aging, additional reliable, independent estimators for the rate of aging are warranted. Urinary biomarkers of oxidative stress and aging could be combined with a number of validated biomarkers of aging, including an array of circulating and/or urinary protein, lipid, exosomal, metabolomic, transcriptomic, and/or epigenetic biomarkers. It is likely that different sets of biomarkers are needed to predict mortality, healthspan, and longevity [46]. Longitudinal studies with large sample sizes and clinically relevant endpoints would help validating the proposed biomarkers of aging and measures of BA in terms of their predictive values for healthspan, longevity, and morbidity of age-related diseases. Lifespan, healthspan, and mortality are determined by many factors, including environmental and lifestyle factors as well genetic factors. The effect of these factors on urinary biomarkers of oxidative stress should also be investigated.

Taken together, our findings suggest that the use of urinary oxidative stress biomarkers can be an important approach for the evaluation of biological age by identifying individuals at accelerated risk for the development of age-related diseases. Testing this hypothesis in future longitudinal studies is thus warranted, given that it cannot be confirmed with a cross-sectional design such as the currently presented work. The use of urinary biomarkers may also provide an easy, robust, and noninvasive tool for evaluation of the effect of antiaging interventions aimed at reducing age-associated inflammatory responses and oxidative stress in prospective cohort studies.

## Figures and Tables

**Table 1:** Characteristics of the participants. For each age group, distribution of males, females, ex-smokers, and never-smokers are shown.

Age groups	Female nonsmoker	Female ex-smoker	Male nonsmoker	Male ex-smoker	BMI
20-29 y. o.	10	1	10	5	24.7 ± 3.0
30-44 y. o.	7	2	10	5	25.4 ± 3.1
45-59 y. o.	6	2	5	6	25.9 ± 2.2
61-75 y. o.	5	3	9	5	25.0 ± 2.4
61-70 y. o.	4	1	6	15	25.6 ± 2.6
71-80 y. o.	6	5	0	3	23.8 ± 3.6

**Table 2:** Regression coefficients and their significance: ∗ indicates *p* < 0.05. Each column represents a model for each response (dependent) variable of interest, while model terms (categorical/continuous predictors) are listed in rows, *n* = 131.

Model term	8-oxoG	8-OHdG	DTyr	iPF2a-VI
Chronological age	0.0048	0.0094	0.0035	0.0260
Sex	0.0358	-0.0026	-0.0418	1.357
Smoking history	0.2736	0.2051	-0.2002	-0.1714
BMI	-0.0001	0.0229	0.0075	0.1084∗
CA ∗ sex	-0.0104∗	0.0006	-0.0025	-0.0101
CA ∗ smoker	0.0077	0.0007	0.0035	0.0087
CA ∗ BMI	0.0001	-0.0003	≈0	-0.0013
Sex ∗ smoker	-0.0567	-0.0726	0.0607	0.1495
Sex ∗ BMI	0.0162	-0.0026	-0.004	-0.0500
Smoker ∗ BMI	-0.0262	-0.0103	-0.009	-0.0107
Power (Cohen's *f*^2^ for effect size)	0.94 (0.09)	0.52 (0.03)	0.96 (0.11)	0.99 (0.26)

**Table 3:** Confusion matrix and performance of the decision tree.

		True labels		Predictive values (PV)
		*A*	*N*	
Predicted labels	A	74	9	Positive PV: 98.7%
	N	1	47	Negative PV: 83.9%
True *A*/*N* rates		Sensitivity: 98.7%	Specificity: 83.9%	Accuracy: 92.3%

## Data Availability

All pertinent data are included either in the main text or in the Supplementary Material.

## Appendix

library(readxl).

library(tidyverse).

library(WGCNA).

library(xlsx).

library(writexl).

dir_project <- “D:/Urinary_Oxidative_Stress/”.

setwd(dir_project).

knitr::opts_knit$set(root.dir = dir_project).

male_biomarker <- read_excel(“Male_UOS_data.xls”).

female_biomarker <- read_excel(“Female_UOS_data.xls”).

bm_pc_m < -prcomp(male_biomarker[,37 : 40], center = TRUE, scale. =TRUE).

bm_pc_f < -prcomp(female_biomarker[,37 : 40], center = TRUE, scale. =TRUE).

bm_pc_m_mat <-bm_pc_m$x.

bm_pc_f_mat <-bm_pc_f$x.

kdm_m < - TrueTrait(datX = bm_pc_m_mat, y = male_biomarker$Age, corFnc = “bicor”, corOptions =“use = ‘pairwise.complete.obs'“).

kdm_m_ba <-kdm_m$datEstimates.

kdm_f < - TrueTrait(datX = bm_pc_f_mat, y = female_biomarker$Age, corFnc = “bicor”, corOptions =“use = ‘pairwise.complete.obs'“).

kdm_f_ba <-kdm_f$datEstimates.

ba_female_data <- cbind(data.frame(female_biomarker$ID, female_biomarker$Age), kdm_f_ba, female_biomarker).

ba_male_data <- cbind(data.frame(male_biomarker$ID, male_biomarker$Age), kdm_m_ba, male_biomarker).

write_xlsx(ba_female_data, “BiolAge_female.xlsx”).

write_xlsx(ba_male_data, “BiolAge_male.xlsx”).

## Supplementary Materials

Supplementary Materials Laboratory parameters of human fresh and frozen serum samples and urine samples (*n* = 198) including urinary oxidative stress parameters.
